# Christ-Siemens-Touraine Syndrome: A Case Report and Review of the Literature

**DOI:** 10.1155/2012/586418

**Published:** 2012-11-29

**Authors:** Sepideh Mokhtari, Saeedeh Mokhtari, Ali Lotfi

**Affiliations:** ^1^Department of Oral and Maxillofacial Pathology, Dental School of Shahid Beheshti University of Medical Sciences, Tehran 4739, Iran; ^2^Department of of Pediatric Dentistry, Dental School of Shahid Beheshti University of Medical Sciences, Tehran 4739, Iran

## Abstract

Ectodermal dysplasia is a rare disorder with defects in two or more of the following structures: the teeth and the skin and its appendages including hair, nails, eccrine, and sebaceous glands. Anhidrotic ectodermal dysplasia is the most common type of disease. This rare disorder, also known as Christ-Siemens-Touraine syndrome, manifests as a triad of hypotrichosis, asteatosis, and anhidrosis. In view of the rarity of this entity, a classical case of anhidrotic ectodermal dysplasia is reported. We have also provided a review of recent investigations in the area of dental abnormalities in this syndrome.

## 1. Introduction

Ectodermal dysplasia (ED) is a genetic disorder that has congenital birth defects of two or more ectodermal structures. Ectodermal dysplasia is divided into two types based on the number and function of sweat glands: hidrotic ectodermal dysplasia (Clouston syndrome) and hypohidrotic (anhidrotic) ectodermal dysplasia (Christ-Siemens-Touraine syndrome) [1]. Hypohidrotic ectodermal dysplasia (HED) is an X-linked condition and is the most common form of ED. In this type of syndrome, there are no sweat glands or they are significantly decreased. HED (Christ-Siemens-Touraine syndrome) has characteristic triad of reduction in the amount of hair (hypotrichosis), absence of sebaceous glands (asteatosis), and absence of sweat glands (anhidrosis) [2]. Some cases are mild, whereas some others are severe. 

Patients often show these manifestations in their appearance: depressed nasal bridge, protuberant lips, prominent supraorbital ridges, sunken cheeks, and wrinkled hyperpigmented skin around the eyes. The oral manifestations include conical or peg-shaped teeth, hypodontia or complete anodontia in both deciduous and permanent dentition, malformation of present teeth, generalized spacing, delayed eruption of permanent teeth, underdeveloped alveolar ridges, and high palatal arch or a cleft palate. Hypoplasia of salivary glands and the absence of oral accessory glands are also present and result in xerostomia and dry cracked lips [1, 3]. In view of the rarity of this syndrome, here, a classical case of anhidrotic ectodermal dysplasia is reported. We have also provided a review of recent investigations in the area of dental abnormalities in this syndrome.

## 2. Case Presentation

**Figure 1:**
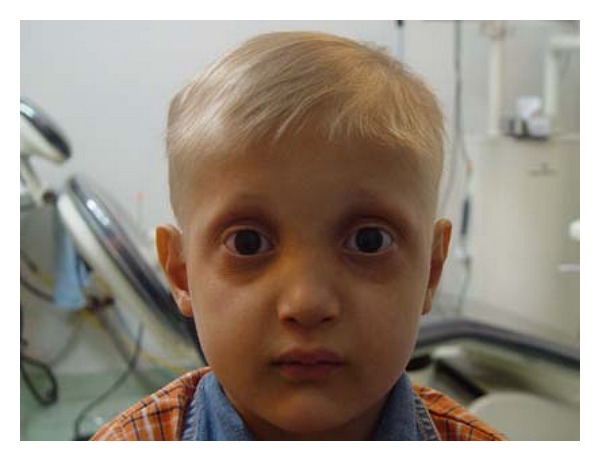
Clinical examination of the patient reveals fine sparse scalp hair, eyebrows, and eyelashes with wrinkled hyperpigmented skin around the eyes.

**Figure 2:**
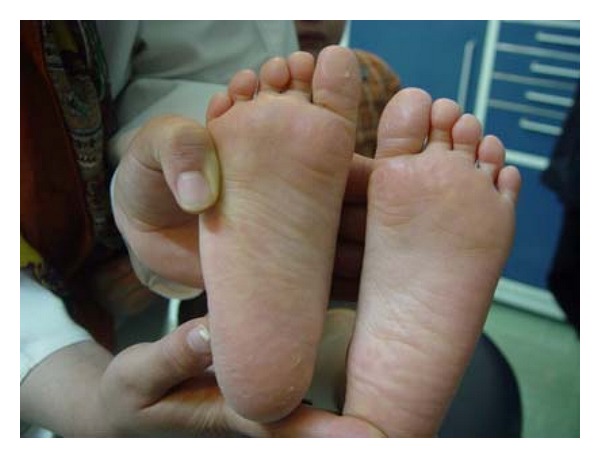
Plantar dyskeratosis is present.

**Figure 3:**
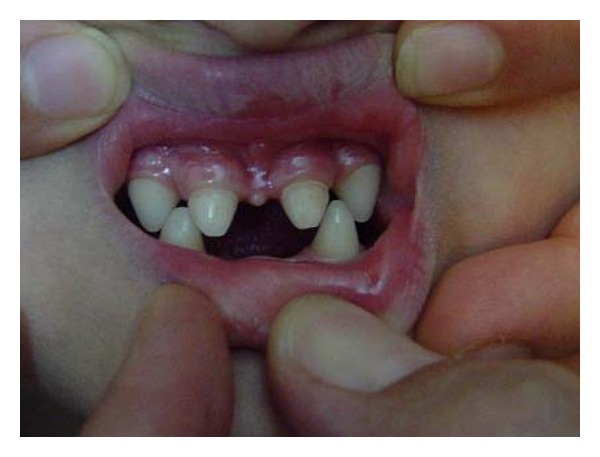
Teeth are reduced in number and are conical in shape.

**Figure 4:**
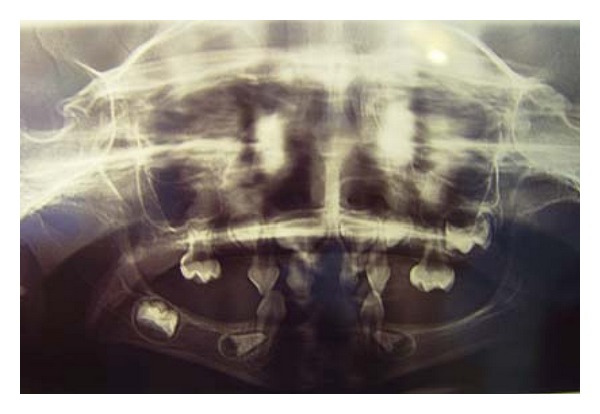
Panoramic radiograph shows eight erupted teeth of primary dentition and eight tooth buds.

A 7-year old boy presented with the chief complaint of difficulty in mastication and lack of esthetics. On general examination, he had sparse, thin, light, blond hair over the scalp, scanty eyebrow and eyelashes, depressed nasal bridge, frontal bossing, and prominent supraorbital ridges. Lips were dry, everted, and prominent. The skin was dry and wrinkled. Hyper pigmentation was evident around the eyes (Figure 1), and plantar dyskeratosis was also present (Figure 2). Oral examination revealed partial anodontia, hypoplastic conical teeth, and delayed eruption of permanent dentition (Figure 3). Panoramic radiograph showed four cone-shaped crowns suggestive of primary canines. Maxillary primary molars and incisors were also present. There was some evidence of tooth formation in both jaws and eight tooth buds probably related to the permanent canines, and first molars and maxillary incisors were present. However, hypodontia in the right and left side was asymmetric, and mandible had more missing teeth than maxilla (Figure 4). He had no nail dystrophy, and his intelligence was normal. His mother stated that her child had recurrent episodes of unexplained hyperpyrexia and thirst; he was not able to sweat, and she had to apply some precautions to protect him from overheating during physical exertion or warm weather. The child was born of nonconsanguineous marriage. Nobody in his family had similar presentations. The child was diagnosed as a case of Christ-Siemens-Touraine syndrome. However, he did not accept to undergo any genetic testing. We considered a removable partial denture as the best treatment option for the child, and so he was referred to a prosthodontist to receive professional prosthodontic procedures.

## 3. Discussion

This patient was a case of Christ-Siemens-Touraine syndrome with the typical presentation of disease in his face and oral cavity. Hypohidrotic ED (Christ-Siemens-Touraine syndrome) is the most frequent form of ectodermal dysplasia, and genetic defects in ectodysplasin signal transduction pathways are the basis of this syndrome. Epithelial cells in hair follicles, eccrine sweat glands, and developing teeth use this pathway during morphogenesis. Therefore, genetic defects result in aplasia, hypoplasia, or dysplasia of these structures. They also lead to disturbances in the enamel matrix and tooth buds and subsequent hypodontia and hypoplasia of teeth [2]. Mutations in the gene Ectodysplasin-A (EDA) underlie X-linked recessive inheritance, which is the most common form of HED. Mutations in the gene Ectodysplasin-A receptor (EDAR) and Ectodysplasin-A Receptor-Associated Adapter (EDARADD) are also responsible for the rare autosomal dominant and autosomal recessive forms [1, 2].

The X-linked recessive HED has full expression in males. Female carriers are more than affected men, but they have little or no signs of the condition and their clinical identification is difficult. However, some investigations have shown that the heterozygous females have a significantly high frequency of agenesis of permanent teeth. They also present an increased prevalence of tooth malformations and reduced tooth size, especially in the mesiodistal dimension [3].

Recently, Zhang et al. have described some correlations between the phenotypes and genotypes of X-linked HED and nonsyndromic hypodontia subjects. They suggested that nonsyndromic hypodontia is probably a variable expression of X-linked HED [4]. Significant differences in the number of primary missing teeth have been detected between X-Linked HED and autosomal recessive HED in recent investigations [5].

Congenital missing of third molars and maxillary lateral incisors is the most common missing. Complete anodontia of both deciduous and permanent dentition is rarely reported. Açikgöz et al. reported the absence of all primary and permanent teeth except the bilaterally unerupted maxillary permanent canines. This shows that the permanent tooth can develop in the absence of its predecessor [6]. Many HED patients also exhibit a relative enlargement of the pulp with various degrees of taurodontism [7].

Even in the case of complete anodontia, the general facial growth pattern is normal in these children. This implies that the development of the jaw does not depend to the presence of teeth. Nevertheless, the alveolar process does not develop in the absence of teeth, and the vertical dimension is reduced, which explains the protuberant everted appearance of lips in these patients [1]. In this case, missing teeth were more in the mandible than in the maxilla. Therefore, the underdevelopment of alveolar process in mandible was more evident in clinical and radiographic features.

Alterations in bone structure, such as hyperdensity of medullary bone, have been found in mandibular symphysis area and other jaw locations of these patients. Although changes in the alveolar bone can be related to oligodontia, changes in the bone structure seem to be tooth independent and suggest a direct effect of genetic defect on bone formation and/or remodeling in this syndrome [1].

If hypohidrosis is managed appropriately, the prognosis for most patients will be very good. Treatment of these children is protecting them from high temperature. In most cases, the preferred treatment option for dental disorders is a removable partial denture, which could also be associated with direct composite restorations. This allows the child to have adequate nutrition, normal appearance, and speech with significant psychosocial benefits. When child reaches teenage years, orthodontic treatment will prepare the mouth for a fixed partial denture or implants in future. In some patients, alveolar ridges are severely hypotrophic due to oligo- or anodontia. This can seriously affect a young person physically and psychologically. In these cases, augmentation of jaws by the use of bicortical corticocancellous bone blocks from hip and delayed implant placement seems to be a suitable treatment option [8]. Immediate loading has also been applied successfully in patients with ectodermal dysplasia [9]. Anesthetic problem is rarely reported in this syndrome [10]. 

Dentists are often the first who diagnose these patients. Therefore, they should be aware of the clinical manifestations of this syndrome. This will be helpful in proper diagnosis, early interventions, and appropriate therapies for these patients.
